# Establishment of a Simple and Rapid Identification Method for *Listeria* spp. by Using High-Resolution Melting Analysis, and Its Application in Food Industry

**DOI:** 10.1371/journal.pone.0099223

**Published:** 2014-06-11

**Authors:** Chihiro Ohshima, Hajime Takahashi, Chirapiphat Phraephaisarn, Mongkol Vesaratchavest, Suwimon Keeratipibul, Takashi Kuda, Bon Kimura

**Affiliations:** 1 Department of Food Science and Technology, Faculty of Marine Science, Tokyo University of Marine Science and Technology, Minato, Tokyo, Japan; 2 Research and Development Center of BETAGRO Group, Klong Luang, Pathumthani, Thailand; 3 Program in Biotechnology, Faculty of Science, Chulalongkorn University, Bangkok, Thailand; 4 Department of Food Technology, Faculty of Science, Chulalongkorn University, Bangkok, Thailand; Cornell University, United States of America

## Abstract

*Listeria monocytogenes* is the causative bacteria of listeriosis, which has a higher mortality rate than that of other causes of food poisoning. *Listeria* spp., of which *L. monocytogenes* is a member, have been isolated from food and manufacturing environments. Several methods have been published for identifying *Listeria* spp.; however, many of the methods cannot identify newly categorized *Listeria* spp. Additionally, they are often not suitable for the food industry, owing to their complexity, cost, or time consumption. Recently, high-resolution melting analysis (HRMA), which exploits DNA-sequence differences, has received attention as a simple and quick genomic typing method. In the present study, a new method for the simple, rapid, and low-cost identification of *Listeria* spp. has been presented using the genes *rarA* and *ldh* as targets for HRMA. DNA sequences of 9 *Listeria* species were first compared, and polymorphisms were identified for each species for primer design. Species specificity of each HRM curve pattern was estimated using type strains of all the species. Among the 9 species, 7 were identified by HRMA using *rarA* gene, including 3 new species. The remaining 2 species were identified by HRMA of *ldh* gene. The newly developed HRMA method was then used to assess *Listeria* isolates from the food industry, and the method efficiency was compared to that of identification by 16S rDNA sequence analysis. The 2 methods were in coherence for 92.6% of the samples, demonstrating the high accuracy of HRMA. The time required for identifying *Listeria* spp. was substantially low, and the process was considerably simplified, providing a useful and precise method for processing multiple samples per day. Our newly developed method for identifying *Listeria* spp. is highly valuable; its use is not limited to the food industry, and it can be used for the isolates from the natural environment.

## Introduction

*Listeria* spp. is a non-sporulating, gram-positive bacterium [1]. The *Listeria* genus consists of *L. monocytogenes*, *L. innocua*, *L. seeligeri*, *L. ivanovii*, *L. grayi*, and *L. welshimeri* [1], as well as the recently classified *L. marthii* [2], *L. rocourtiae* [3], *L. fleischmannii* [4], and *L. weihenstephanensis* [5]. *Listeria* spp. are widely present throughout the natural world, and has been isolated from livestock, soil, plants, river water, silage [6], and marine products [7].

Of the *Listeria* species, *L. monocytogenes* can be transmitted among humans and animals, and it is the cause of listeriosis [1]. It can also cause sporadic food poisoning–for which it is known in various Western countries [8],[9]–through milk products such as cheese [9] and processed meats such as sausages and salami [10]. In the U.S.A., FDA standards have zero tolerance for *L. monocytogenes* contamination in processed foods, but with certain exceptions [11]. Likewise, the contamination level of *L. monocytogenes* in processed foods is strictly regulated in the EU at <100 cfu/g [12]. Strict contamination management for *L. monocytogenes* is therefore necessary at food processing plants, and testing is carried out at multiple points in the manufacturing process [9],[13]. *L. innocua*, which is thought to behave in a manner similar to *L. monocytogenes*, is also included [14]. Additionally, to analyze the route of contamination of food products, environments such as farms [9],[15] and fish farms [16] have been examined.

In the food industry, the FDA Bacteriological and Analytical Method (BAM) and the International Organization of Standards (ISO) 11290 method are used for detecting *Listeria* spp. [1]. In both methods, culturing in a liquid culture medium containing a selective agent is followed by the isolation of typical colonies and their culturing on selective media such as Oxford or PALCAM. Api Listeria [17], 16S rDNA sequence [18], multiplex PCR [19], and multilocus sequence typing (MLST) [19] have all been used to identify *Listeria* spp. isolated by these methods. Api Listeria does not detect all *Listeria* spp., thus making it unsuitable for precise identification. Methods that use sequence analysis, such as 16S rDNA sequencing and MLST, have high accuracy and reproducibility; however, they are complicated and expensive [21], making them unsuitable for evaluating large quantities of samples [1]. Rapid testing is critical for the food industry, and it is necessary that the methods be inexpensive and relatively easy to perform.

High-resolution melting analysis (HRMA) utilizes the different temperatures at which the double-stranded DNA is dissociated. The procedure is simple and has gained attention for its usefulness in to large- scale testing. The time required for HRMA following PCR amplification is approximately 1 hour at maximum, which results in relatively reduced time required for identification [21]. To date, HRMA-based methods have been developed for the identification and typing of *Cronobacter* spp. in milk [21], and for identifying well-known serotypes of *Salmonella* [22]. This technique has received a lot of attention in fields other than food microbiology; by using HRMA for examining specific genes, methods have been developed to identify the other ingredient oil which is mixed with olive oil [23], as well as hookworm infection in humans [24].

Wang et al. [25] and Jin et al. [26] used HRMA in the identification of *Listeria* spp. In the Wang et al. study, the intergenic spacer region of the rRNA was used as the target region, and *ssrA* was used by Jin et al. Neither method investigated the recently reported *L. fleischmannii*, *L. rocourtiae*, or *L. marthii*, and it is unknown whether conventional methods could be used to identify all the currently registered *Listeria* spp. As a preliminary experiment, the method by Jin et al. was used for *L. fleischmannii* and *L. rocourtiae*, but it could not identify the 2 species. *L. fleischmannii* and *L. rocourtiae* have both been reportedly isolated from food [3],[4], and the usefulness of methods that cannot identify the 2 species is limited, especially in the food industry. Therefore, we developed a method for identifying all the 9 *Listeria* species by using a novel gene target, and evaluated the new method by using it for analyzing bacterial isolates from the food industry.

## Materials and Methods

### Strains

Strains used in this study are listed in Table 1. Thirteen strains of 9 *Listeria* spp. were obtained from American Type Culture Collection (ATCC), Deutsche Sammlung von Mikroorganismen und Zellkulturen GmbH (DSMZ), Collection of Institute Pasteur (CIP) and 6 strains of 3 *Listeria* spp. were isolated from a food processing plant or the environment. One strain of *L. seeligeri* used in this study was isolated from the river water located in Hokkaido, Japan(140.15596, 42.29360). The sampling site is located in open access area and no specific permissions are required to collect samples. Additionally, endangered or protected species were not collected.

**Table 1:** Bacterial strains used in this study.

Species	Strain no.
*Listeria monocytogenes*	ATCC19114
	ATCC19115
	ATCC19116
	CIP103575(SottA)
	CIP107776(EGDe)
*Listeria innocua*	ATC33090^T^
	1-2
	8-1
	1-25
	26-1
*Listeria sseligeri*	ATCC35967^T^
	12.9.11.2-1
*Listeria rocouriae*	DSM22097
*Listeria ivanovii*	ATCC19119^T^
*Listeria grayi*	ATCC19120^T^
*Listeria welshimeri*	ATCC35897^T^
	019-3w
*Listeria marthii*	DSM23913
*Listeria fleischmannii*	DSM24998

### DNA Extraction

Strains were grown in Trypticase Soy Broth (TSB) (Becton Dickinson, U.S.A.) overnight at 37°C. Bacterial cells were harvested from 1 mL TSB by centrifugation at 8,000×*g* for 3 min and the supernatant was removed. Total genomic DNA was extracted using NucleoSpin Tissue (Macherey Nagel, Germany) according to the manufacturer’s protocol.

### Primer Design

The *rarA*, which encodes a recombination factor protein, and the *ldh*, which encodes L-lactate dehydrogenase, were chosen as target genes [20]. Sequence date for the *rarA* of *L. monocytogenes* strain FSL S4-465 (GenBank accession number: GU475922.1), *L. innocua* strain FSL R6-556 (GU475917.1), *L. seeligeri* strain FSL S4-009 (GU475926.1), *L. rocourtiae* strain CIP 109804 (JQ287768.1), *L. ivanovii* subsp. ivanovii strain FSL F6-600 (GU475932.1), *L. grayi* DSM 20601 (CCR02000005.1), *L. welshimeri* strain FSL S4-182 (GU475919.1), *L. fleischmannii* LU2006-1 c28 (ALWW01000009.1), *L. marthii* strain FSL S4-120(GU475909.1), and sequence date for the *ldh* of *L. monocytogenes* strain EGDe (AL591824.1), *L. innocua* Clip11262 (NC_003212.1), *L. seeligeri* strain FSL S4-171 (GU475600.1), *L. ivanovii* subsp. ivanovii PAM 55 (NC_016011.1), *L. grayi* DSM 20601 (ACCR02000005.1), *L. welshimeri* serovar 6b strain SLCC5334 (NC_008555.1), *L. fleischmannii* LU2006-1 c9 (NZ_ALWW01000007.1), and *L. marthii* strain FSL S4-120 (GU475572.1) were obtained from GenBank. Alignment was performed using the Genetyx-Win program (Software Development Co., Japan). Unique and specific primer pairs for *rarA* and *ldh* were developed using the above information (Table 2).

**Table 2:** Sequence of primers used in this study.

Forward primer	Sequence (5′→3′)	Reverse primer	Sequence (5′→3′)
For *rarA* amplification			
rarA-f universal	GGYGCVACRACDAGTAATCC	rarA-r universal	CCRTTRCTSGCHGTTGC
rarA-f L.grayi	CGCTACCACCAGTAATCC	rarA-r L.rocourtiae/seeligeri	CCATTACTCGCMGTCGC
rarA-f L.innocua	GCAACGACGAGTAACCC	rarA-r L.innocua/ivanovii	CCRTTACTYGCYGTGGCAA
rarA-f L.fleischmannii	GCCACAACGAGCAATCC	rarA-r L.fleischmannii	CCACCACTTGCTGTCGC
For *ldh* amplification			
ldh-f	GGYAAAATCGCATTTTCGTTA	ldh-r	CCAGCWTGGAGCCAYACAAC

### PCR Amplification of *rarA* Gene and *ldh* Gene

Partial *rarA* and *ldh* gene fragments were amplified for the 19 bacterial strains listed in Table 1. PCR was performed in a final volume of 50 µL. The PCR reaction mix for *rarA* contained 10 mM Tris-HCl (pH 8.3), 50 mM KCl, 1.5 mM MgCl_2_, 0.2 mM of each dNTP, 500 nM rarA-f universal primer, 167 nM rarA-f L. grayi primer, 167 nM rarA-f L. innocua primer, 167 nM rarA-f L. fleischmannii primer, 375 nM rarA-r universal primer, 250 nM rarA-r L. rocourtiae/seeligeri primer, 250 nM rarA-r L. innocua/ivanovii primer, 125 nM rarA-r L. fleischmannii primer, 25 ng template DNA, and 0.5 U Takara Taq DNA polymerase (Takara Bio, Japan). The PCR reaction mix of *ldh* contained 1 µM ldh-f and ldh-r primers instead of *rarA* primers. Primer sequences used in this study are shown in Table 2. Amplification was performed using the GeneAmp PCR System 9700 thermalcycler (Life Technologies, U.S.A.). The following parameters ware used for amplifying *rarA*: 95°C for 5 min, 35 cycles of 95°C for 10 s, 56°C for 30 s, 72°C for 30 s, and 72°C for 1 min. For amplification of *ldh*, the following conditions were used: 95°C for 5 min, 35 cycles of 95°C for 10 s, 60°C for 30 s, and 72°C for 1 min. PCR products were confirmed by electrophoresis in a 2% agarose gel.

### HRMA

Following confirmation of target gene amplification, 1 µL of 20× Resolight Dye (Roche, Germany) was added to 19 µL of the PCR reaction, and HRMA was carried out with a LightCycler 480 at 75 acquisitions/°C with the following steps: 95°C for 1 min and 40°C for 1 min, followed by increasing the temperature from 60 to 99°C at 0.01°C/s.

For *rarA* analysis, Light Cycler 480 gene scanning program (Roche) was used. The straight-line parts of the DNA dissociation curve at the time of dissociation (73.06–79.05°C and 90.09–91.77°C) were selected for normalization, and differences in the shape of the normalized and temperature-shifted plots according to each *Listeria* spp. were determined. The melting peak of *L. monocytogenes* CIP103575 was used as a baseline control. For *ldh* analysis of *L. monocytogenes* and *L. welshimeri,* Tm calling was performed using the Light Cycler 480 software and the Tm value of the amplification products determined. All samples of *ldh* were examined in triplicate and obtained the standard deviation (SD) for the Tm value.

### Application to Actual Food Factory Isolates

Developed methods of HRMA targeting *rarA* and *ldh* genes was confirmed using *Listeria* spp. strains obtained from food industry isolates. Eighty one isolates from the food industry, as well as *L. monocytogenes* CIP103575, were used. The preserved strains were grown in TSB medium and cultured at 30°C overnight. After culturing, 1 mL of bacterial suspension was centrifuged and the pellet obtained. Using a genomic DNA extraction kit (RBC Bio Science, Taiwan), DNA was extracted from the pellet according to the manufacturer’s protocol. Experiments were then performed as described.

To confirm the results of HRMA, identification by 16S rDNA sequencing was also performed. Bacterial 16S rDNA genes were amplified by PCR using the following universal primers: 27F (5′-AGA GTT TGA TCC TGG CTC AG-3′) and 1492R (5′-GGT TAC CTT GTT ACG ACT T-3′) [27]. PCR was performed in a final reaction volume of 50 µL consisting of 10 mM Tris-HCl (pH 8.3), 50 mM KCl, 1.5 mM MgCl_2_, 1 µM of each primer, 0.2 mM of each dNTP, 25 ng template DNA, and 0.5 U Takara Taq DNA polymerase (Takara Bio). The GeneAmp PCR System 9700 thermocycler (Life Technologies) was used to amplify the target products. Cycling conditions were as follows: 94°C for 1 min, 35 cycles of 94°C for 1 min, 58°C for 1 min, 72°C for 1 min, and 72°C for 7 min. Following the reaction, amplification products were purified by Agencourt AMPure (Beckman Coulter, U.S.A), according to the manufacturer’s protocol.

Following purification, the amplification products were sequenced using BigDye Terminator v3.1 Cycle sequencing kit (Life Technologies) according to the manufacturer’s protocol. For the sequencing reaction, in addition to the 27F primer which was used in PCR, the following 2 primers were used: 510F (5′-CAG CMG CCG CGG TAA TAC G-3′), and 907F (5′-AAA CTC AAA KGA ATT GAC GG-3′).

After completion of the sequencing reaction, the sequence determined using an ABI PRISM 3130 Genetic Analyzer (Life Technologies). A consensus region (contig) was resolved by Genetix-ATGC using the sequences from each primer, and compared by BLAST search of the DNA Data Bank of Japan (DDBJ), and a bacterial species assigned where homology was greater than 98%.

## Results

### Establishment of a Method to Identify *Listeria* spp

**Figure 1:**
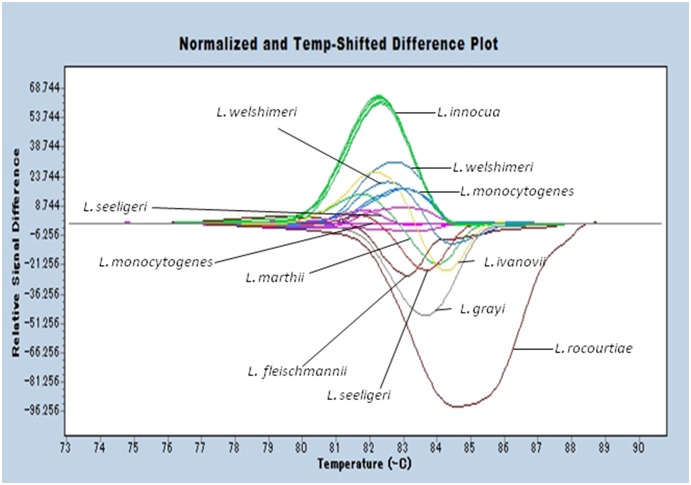
The result of high resolution melting analysis of *rarA* for 19 strains of 9 *Listeria* spp. Representative profiles of the high resolution melting curves (normalized and temperature shifted difference plot) of *rarA* amplicons for *L. innocua* (upper green line), *L. welshimeri* ^T^ (upper blue line), *L. welshimeri* 019-3w (blue line in the middle), *L. monocytogenes* ATCC19114, ATCC19116 (lower blue lines), *L. monocytogenes* CIP107776, CIP103575 (base line), ATCC19115 (pink lines), *L. seeligeri* ^T^ (pink line), *L. fleischmannii* ^T^ (upper brown line), *L. seeligeri* 2–1 (red line), *L. marthii* ^T^ (lower green line), *L. ivanovii* ^T^ (yellow line), *L. grayi* ^T^ (gray line) and *L. rocourtiae* ^T^ (lower brown line). T: type strain.

HRMA was performed on the *rarA* of 19 strains from 9 species of *Listeria* spp. (Figure 1), resulting in the classification of 5 strains of *L. monocytogenes* into 2 patterns. The 5 *L. innocua* strains could be grouped together. *L. seeligeri* isolated from the environment showed a specific pattern; however, typestrain of *L. seeligeri* was grouped with *L. monocytogenes* strains. *L. fleischmannii*, *L. grayi*, *L. ivanovii*, *L. rocourtiae*, and *L. marthii* each showed specific patterns. However, the type strain and the isolated strain from food industry of *L. welshimeri* could not be distinguished from *L. monocytogenes*.

The Tm value was then investigated for the *ldh* of *L. monocytogenes* and *L. welshimeri*, which could not be determined by HRMA of *rarA*. Tm values of the 5 strains of *L. monocytogenes* ranged from 82.57±0.30–83.25±0.32°C, and Tm of the 2 *L. welshimeri* strains were 83.88±0.15 and 84.08±0.12°C, respectively (Table 3). Since the Tm values of the 2 species differed by at least 0.5°C, the Tm values can be used to differentiate between the 2 species. Reproducibility was confirmed by analyzing the Tm values in triplicate.

**Table 3:** Tm value of 5 *L. monoytogenes* strains and 2 *L. welshimeri* strains for *ldh* gene.

species	strain	Tm (°C)
*L. monocytogenes*	ATCC19115	82.91
	ATCC19114	83.22
	CIP103575	83.36
	CIP107776	83.36
	ATCC19116	83.62
*L. welshimeri*	ATCC35897^T^	84.02
	019-3w	84.20

### Application to Actual Food Industry Isolates

**Figure 2:**
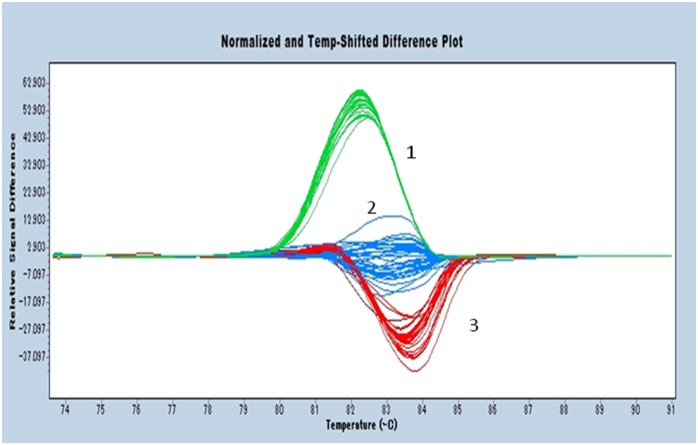
The result of high resolution melting analysis of *rarA* for 81 strains isolated from food processing plant. Representative profiles of the high resolution melting curves (normalized and temperature shifted difference plot) of *rarA* amplicons for 81strains isolated from food processing plant. They were classified for 1: *L. innocua*, 2: *L. monocytogenes*/*L. welshimeri* and 3: *L. seeligeri* on the basis of HRM curve profiles. One strain colored by brown was unidentified.

To evaluate the newly established method of HRMA, identification of 81 strains isolated from the food-processing plant was performed. HRM peak patterns of *rarA* were classified broadly into 3 groups. Twenty-one were *L. innocua*, and 26 were *L. seeligeri*, while 33 strains were classified into the *L. monocytogenes*/*L. welshimeri* group (Figure 2). The peak pattern of 1 strain did not fit into any group. The 33 strains classified into the *L. monocytogenes*/*L. welshimeri* group underwent species identification using *ldh*. Using the previously described method, strains with a Tm value of 83.31°C or below were designated *L. monocytogenes*, and those with a Tm higher than 83.82°C designated *L. welshimeri*, resulting in 18 identified as *L. welshimeri* and 14 as *L. monocytogenes*, with 1 strain remaining unidentified (Table 4).

**Table 4:** Tm value of 33 strains which coud not identify by *rarA* gene HRMA.

Strain no.	Tm (°C)	判定結果
31	83.06	*monocytogenes*
21	83.1	*monocytogenes*
76	83.12	*monocytogenes*
35	83.12	*monocytogenes*
23	83.12	*monocytogenes*
52	83.13	*monocytogenes*
12	83.13	*monocytogenes*
20	83.17	*monocytogenes*
11	83.18	*monocytogenes*
28	83.19	*monocytogenes*
22	83.2	*monocytogenes*
18	83.21	*monocytogenes*
25	83.21	*monocytogenes*
19	83.25	*monocytogenes*
46	83.64	*monocytogenes*
6	83.95	could not identify
13	84.04	*welshimeri*
16	84.12	*welshimeri*
2	84.14	*welshimeri*
61	84.2	*welshimeri*
4	84.2	*welshimeri*
7	84.2	*welshimeri*
14	84.22	*welshimeri*
10	84.24	*welshimeri*
9	84.25	*welshimeri*
1	84.26	*welshimeri*
38	84.27	*welshimeri*
5	84.3	*welshimeri*
37	84.32	*welshimeri*
17	84.35	*welshimeri*
15	84.42	*welshimeri*
3	84.8	*welshimeri*
45	85.56	*welshimeri*

The results of HRMA identification were validated using 16S rDNA sequencing. The 21 strains identified as *L. innocua* by the HRMA of *rarA* gave the same results in 16S rDNA sequencing. Additionally, the 14 strains of *L. monocytogenes* and 18 strains of *L. welshimeri* that were identified by a combination of *rarA* and *ldh* data also produced the same results in 16S rDNA sequencing. Among the 26 strains identified as *L. seeligeri* from the HRMA of *rarA*, 22 strains were confirmed as *L. seeligeri* by 16S rDNA sequencing. Four strains showed results that were different for 16S rDNA sequencing from those of HRMA; 2 were identified as *L. monocytogenes* from 16S rDNA sequencing, while the other 2 were identified as *L. welshimeri*. Additionally, the strain not belonging to any group from the HRMA of *rarA* was determined to be *L. welshimeri*. The strain for which identification was unfeasible due to an *ldh* Tm value of 83.64°C was also *L. monocytogenes*. The success rate of species identification by HRMA was 100% for *L. monocytogenes*, *L. innocua*, and *L. welshimeri*, and 84.6% for *L. seeligeri* (Table 5). The overall success rate for all 81 strains was 92.6%.

**Table 5:** Comparison of results of identification by HRM and 16S rDNA sequencing Listeria spp. isolated from the food industry.

Listeria spp.	A: Number ofstrains identifiedby HRMA	B: Number of strains inagreement between the HRMAand 16S rDNA sequencingidentificaation results	Success rate of the HRMA identification method (B/A)
*L. monocytogenes*	14	14	100.0%
*L. innocua*	21	21	100.0%
*L. seeligeri*	26	22	84.6%
*L. welshimeri*	18	18	100.0%
could not identify	2	-	-
total	81	75	92.6%

The sequences of primer region are omitted.

## Discussion

In the present study, a method was developed for identifying *Listeria* spp. via HRMA of the polymorphic regions in *rarA* and *ldh*. In an evaluation of 9 species–*L. monocytogenes*, *L. innocua*, *L. seeligeri*, *L. rocourtiae*, *L. ivanovii*, *L. grayi*, *L. welshimeri*, *L. marthii*, and *L. fleischmannii*–by using the newly developed HRMA method, 7 species showed an intrinsic peak pattern following the HRMA of *rarA*, and identification of the remaining 2 species was possible by analysis of the *ldh* Tm values. The *rarA* sequences of *Listeria* strains which were compared for designing the primer sets and were used in Figure 1 were aligned to confirm the variety of HRM profile of each *Listeria* spp.(Figure S1). The *ldh* sequences of *L. monocytogenes* and *L. welshimeri* were also aligned (Figure S2). It was considered that the polymorphism of these sequences which were different from species to species showed diverse HRM peak patterns and Tm values.

There were 2 issues that arose with the initial attempt to identify *Listeria* spp. by using *rarA* alone. First, the HRM peak pattern differed between the type strain of *L. seeligeri* and the environmental isolate of *L. seeligeri*. The environmental isolate of *L. seeligeri* showed a completely different specific peak compared to other species, while the type strain showed the same peak as that of *L. monocytogenes*. To determine the reason for this difference, the sequences of *rarA* regions of the 2 *L. seeligeri* strains and *L. monocytogenes* CIP103575 were compared. The sequences of the 2 strains of *L. seeligeri* differed by 3 out of 202 bases; therefore, even within the same species, the sequence is not identical. Furthermore, 34 differences in the sequence were found between the type strain of *L. seeligeri* and *L. monocytogenes* CIP103575, but because the respective GC content were 41.1% and 41.6%, both species produced similar Tm values. HRMA uses the differences in GC content, the composition of bases, and sequence lengths [24]. In this case, although the sequence differed, the GC content was very similar across the species, thereby producing a common HRMA peak pattern. The second issue was that *L. monocytogenes* and *L. welshimeri* could not be distinguished by using the HRMA peak pattern of *rarA* alone. Comparison of the sequences of the *rarA* region used in HRMA of *L. monocytogenes* and *L. welshimeri* showed that 35 out of 200 base pairs were different. However, as with *L. seeligeri*, discrimination was difficult due to similar GC levels (41.6 and 41.4%, respectively). Pietzka et al. investigated the relationship between mutations, single nucleotide polymorphisms (SNPs), and HRMA curves, and demonstrated that even if the sequence was different, the same melting curve results if the Tm values were similar [28]. In this study, strains that could not be distinguished were those with similar Tm values.

Identification using *ldh* was attempted for the 2 strains that could not be distinguished by *rarA* HRMA. The results of *ldh* HRMA of 5 *L. monocytogenes* strains, as well as the 2 strains of *L. welshimeri*, are shown in Table 2. The Tm values of the 2 species were very different, and thus criteria could be established for their identification.

Evaluation of the newly developed method on the isolates from the food processing plant showed an exceptionally high identification rate of 92.6%. As described previously, *L. seeligeri* was problematic in that it exhibited 2 peak patterns in HRMA of *rarA*; however, for identification of food industry isolates the present method had a high success rate of 84.6% for *L. seeligeri*, and all strains identified as *L. seeligeri* by 16S rDNA sequencing were also identified as such by HRMA. From these results, it is likely a non-issue for actual daily testing in factory. The typestrain of *L. seeligeli* was thought to have untypical *rarA* sequence, because the 22 strains of *L. seeligeri* isolated from food industry showed same HRM profile as the isolated strain *L. seeligeri* 2–1. Additionally, differentiation between the 2 strains by Tm value analysis of *ldh* on isolates showed that out of 33 strains, 32 were correctly identified, demonstrating the practicality of the criteria in the present study.

The method reported by Wang et al. [25] shows an HRM peak pattern with 2 peaks, and identification of species are based on a combination of each peak. Consequently, each *Listeria* spp. has multiple patterns. In the method established in the present study, there was generally 1 peak pattern per species of *Listeria*, and it has advantages of simple waveform determination. A subsequent analysis using *ldh* is necessary only where a strain is thought to be *L. monocytogenes* or *L. welshimeri*, and since it only requires the analysis of Tm values, it is simple to perform.

Jin et al. [26] used *ssrA* as a target, and exploited the differences in Tm values for each *Listeria* spp.; however, the Tm values were very close between each strain (83.51–86.12°C). In the present study, identification was performed using the shape of the melt curve, and the Tm values for the products were not close, with a range of 82.28–88.66°C. The Tm values also influence the melt curve, and if the present method is compared to previous ones, differences between Tm values of different strains are clear, resulting in a more accurate identification. Additionally, because the differences in Tm values were large, a Light Cycler nano–designed for easy implementation of HRMA, but which was less precise than the Light Cycler 480–could be used to further simplify the process. Since the instrument is comparatively low-priced, it would be easier to introduce into research institutes.

In the present study, a method was established using HRMA of *rarA* and *ldh*, which identified 9 species belonging to the genus *Listeria*. The food industry uses FDA BAM and ISO methods for testing food products for *Listeria*, and if typical colonies are confirmed on a selective culture medium, species identification of the strain is necessary. Since strain identification can take several additional days, the present method, which needs only hours, can contribute significantly to increasing the rapidity of testing. The present study assumes the HRMA is carried out on pure, isolated colonies, and would be easy and appropriate to adopt for daily testing carried out by food companies. The method can be considered sufficiently applicable, as evaluation on actual isolates from the food factory identified *Listeria* spp. with a success rate of 92.6%. In addition to the 9 species used in the present study, *L. weihenstephanensis* has been identified recently as a member of the *Listeria* genus. Based on the present study, the likelihood of isolating this species in the food industry is low; however, it is necessary to have methods to identify such strains of *Listeria* spp. distributed in the environment.

The simplicity and rapidity of HRMA method surpasses that of identification by sequence analysis, and its concurrence with the 16S rDNA sequence analysis was also high. Our newly developed method for identifying *Listeria* spp. is highly valuable; its use is not limited to the food industry, and it can be extended to identifying strains isolated from the natural environment.

## Supporting Information

Figure S1 Alignment of partial sequences of *rarA* gene used for designing primer and analysis. The sequences of primer region are omitted.
(TIF)

Figure S2 Alignment of partial sequences of ldh gene used for designing primer and analysis.
(TIF)

## References

[1] Gasanov U, Hughes D, Hansbro PM (2005) Methods for isolation and identification of *Listeria* spp. and *Listeria monocytogenes* review. FEMS Microbiol Reviews 29: 851–875. 10.1016/j.femsre.2004.12.002 16219509

[2] Graves LM, Helsel LO, Steigerwalt AG, Morney RE, Daneshvar MI, et al (2010) *Listeria marthii* sp. nov., isolated from the natural environment, Finger Lakes National Forest. Int J Syst Evol Microbiol 60: 1280–1288. 19667380 10.1099/ijs.0.014118-0

[3] Leclercq A, Clermont D, Bizet C, Grimont PA, Le Flèche-Matéos A, et al (2010) *Listeria rocourtiae* sp. nov. Int J Syst Evol Microbiol 60: 2210–2214. 19915117 10.1099/ijs.0.017376-0

[4] Bertsch D, Rau J, Eugster MR, Haug MC, Lawson PA, et al (2013) *Listeria fleischmannii* sp. nov., isolated from cheese. Int J Syst Evol Microbiol 63: 526–532. 22523164 10.1099/ijs.0.036947-0

[5] Halter EL, Neuhaus K, Scherer S (2013) *Listeria weihenstephanensis* sp. nov., isolated from the water plant Lemna trisulca taken from a freshwater pond. Int J Syst Evol Microbiol 63: 641–647. 22544790 10.1099/ijs.0.036830-0

[6] Vongkamjan K, Switt AM, Bakker HC, Fortes ED, Wiedmann M (2012) Silage collected from dairy farms harbors an abundance of Listeriaphages with considerable host range and genome size diversity. Appl Environ Microbiol 78: 8666–8675. 23042180 10.1128/AEM.01859-12 PMC3502902

[7] Miya S, Takahashi H, Ishikawa T, Fujii T, Kimura B (2010) Risk of *Listeria monocytogenes* contamination of raw ready-to-eat seafood products available at retail outlets in Japan. Appl Environ Microbiol 76: 3383–3386. 20348310 10.1128/AEM.01456-09 PMC2869148

[8] Cartwright EJ, Jackson KA, Johnson SD, Graves LM, Silk BJ, et al (2013) Listeriosis outbreaks and associated food vehicles, United States, 1998–2008. Emerg Infect Dis 19: 1–9. 23260661 10.3201/eid1901.120393 PMC3557980

[9] Fox E, Hunt K, O’Brien M, Jordan K (2011) *Listeria monocytogenes* in Irish farmhouse cheese processing environments. Int J Food Microbiol 145: S39–S45. 21087802 10.1016/j.ijfoodmicro.2010.10.012

[10] Rodríguez-Lázaro D, Jofré A, Aymerich T, Hugas M, Pla M (2004) Rapid quantitative detection of *Listeria monocytogenes* in meat products by real-time PCR. Appl Environ Microbiol 70: 6299–6301. 15466579 10.1128/AEM.70.10.6299-6301.2004 PMC522080

[11] Swaminathan B (2001) *Listeria monocytogenes*. In: Doyle MP,BeuchatLR., Montville TJ., (Eds), Food microbiology, Fundanmentals and Frontiers ASM Press Washington DC: 383–409.

[12] Jadhav S, Bhave M, Palombo EA (2012) Methods used for the detection and subtyping of *Listeria monocytogenes* . J Microbiol Meth 88: 327–341. 10.1016/j.mimet.2012.01.002 22261140

[13] Vogel BF, Huss HH, Ojeniyi B, Ahrens P, Gram L (2001) Elucidation of *Listeria monocytogenes* contamination routes in cold-smoked salmon processing plants detected by DNA-based typing methods. Appl Environ Microbiol 67: 2586–2595. 11375167 10.1128/AEM.67.6.2586-2595.2001 PMC92911

[14] Oliveira M, Usall J, Viñas I, Solsona C, Abadias M (2011) Transfer of *Listeria innocua* from contaminated compost and irrigation water to lettuce leaves. Food Microbiol 28: 590–596. 21356469 10.1016/j.fm.2010.11.004

[15] Strawn LK, Fortes ED, Bihn EA, Nightingale KK, Gröhn YT, et al (2013) Landscape and meteorological factors affecting prevalence of three food-borne pathogens in fruit and vegetable farms. Appl Environ Microbiol 79: 588–600. 23144137 10.1128/AEM.02491-12 PMC3553790

[16] Miettinen H, Wirtanen G (2005) Ecology of *Listeria* spp. in a fish farm and molecular typing of *Listeria monocytogenes* from fish farming and processing companies. Int J Food Microbiol 112: 138–146. 10.1016/j.ijfoodmicro.2006.06.016 16842875

[17] McLauchlin J (1997) The identification of *Listeria* species. Int J Food Microbiol 38: 77–81. 9498140 10.1016/s0168-1605(97)00086-x

[18] Hellberg RS, Martin KG, Keys AL, Haney CJ, Shen Y, et al (2013) 16S rRNA partial gene sequencing for the differentiation and molecular subtyping of *Listeria* Species. Food Microbiol 36: 231–240. 24010602 10.1016/j.fm.2013.06.001

[19] Huang B, Eglezos S, Heron BA, Smith H, Graham T, et al (2007) Comparison of multiplex PCR with conventional biochemical methods for the identification of *Listeria* spp. isolates from food and clinical samples in Queensland, Australia. J Food Prot 70: 1874–1880. 17803144 10.4315/0362-028x-70.8.1874

[20] den Bakker HC, Bundrant BN, Fortes ED, Orsi RH, Wiedmann M (2010) A population genetics-based and phylogenetic approach to understanding the evolution of virulence in the genus *Listeria* . Appl Environ Microbiol 76: 6085–6100. 20656873 10.1128/AEM.00447-10 PMC2937515

[21] Cai XQ, Yu HQ, Ruan ZX, Yang LL, Bai JS, et al (2013) Rapid detection and simultaneous genotyping of *Cronobacter* spp. (formerly *Enterobacter sakazakii*) in powdered infant formula using real-time PCR and high resolution melting (HRM) analysis. PLoS One 8: e67082. 23825624 10.1371/journal.pone.0067082 PMC3692429

[22] Zeinzinger J, Pietzka AT, Stöger A, Kornschober C, Kunert R, et al (2012) One-Step triplex high-resolution melting analysis for rapid identification and simultaneous subtyping of frequently isolated *Salmonella* serovars. Appl Environ Microbiol 78: 3352–3360. 22344662 10.1128/AEM.07668-11 PMC3346493

[23] Ganopoulos I, Bazakos C, Madesis P, Kalaitzis P, Tsaftaris A, et al (2013) Barcode DNA high-resolution melting (Bar-HRM) analysis as a novel close-tubed and accurate tool for olive oil forensic use. J Sci Food Agr 93: 2281–2286. 23400707 10.1002/jsfa.6040

[24] Ngui R, Lim YA, Chua KH (2012) Rapid detection and identification of human hookworm infections through high resolution melting (HRM) analysis. PLoS One 7: e41996. 22844538 10.1371/journal.pone.0041996 PMC3406038

[25] Wang J, Yamada S, Ohashi E (2010) Rapid identification of *Listeria* species and screening for variants by melting curve and high-resolution melting curve analyses of the intergenic spacer region of the rRNA gene. Can J Microbiol 56: 676–682. 20725130 10.1139/w10-054

[26] Jin D, Luo Y, Zhang Z, Fang W, Ye J, et al (2012) Rapid molecular identification of *Listeria* species by use of real-time PCR and high-resolution melting analysis. FEMS Microbiol Lett 330: 72–80. 22372942 10.1111/j.1574-6968.2012.02535.x

[27] Weisburg WG, Barns SM, Pelletier DA, Lane DJ (1991) 16S ribosomal DNA amplification for phylogenetic study. J Bacteriol 173: 697–703. 1987160 10.1128/jb.173.2.697-703.1991 PMC207061

[28] Pietzka AT, Stöger A, Huhulescu S, Allerberger F, Ruppitsch W (2011) Gene Scanning of an Internalin B Gene Fragment Using High-Resolution Melting Curve Analysis as a Tool for Rapid Typing of *Listeria monocytogenes* . J Mol Diagn 13: 57–63. 21227395 10.1016/j.jmoldx.2010.11.002 PMC3069814

