# Correction: Establishment of a Simple and Rapid Identification Method for *Listeria* spp. by Using High-Resolution Melting Analysis, and Its Application in Food Industry

**DOI:** 10.1371/journal.pone.0107342

**Published:** 2014-09-02

**Authors:** The PLOS ONE Staff

There are errors in Tables 1, 3 and 4. Please see the correct tables here.

**Table 1:** Bacterial strains used in this study.

Species	Strain no.
*Listeria monocytogenes*	ATCC19114
	ATCC19115
	ATCC19116
	CIP103575(SottA)
	CIP107776(EGDe)
*Listeria innocua*	ATCC33090^T^
	1-2
	8-1
	1-25
	26-1
*Listeria seeligeri*	ATCC35967^T^
	2-1
*Listeria rocourtiae*	DSM22097
*Listeria ivanovii*	ATCC19119^T^
*Listeria grayi*	ATCC19120^T^
*Listeria welshimeri*	ATCC35897^T^
	019-3w
*Listeria marthii*	DSM23913^T^
*Listeria fleischmannii*	DSM24998^T^

**Table 3:** *Idh* gene Tm value of 5 *L. monoytogenes* strains and 2 *L. welshimeri* strains.

Species	Strain	Tm (°C)
*L. monocytogenes*	ATCC19115	82.57±0.30
	ATCC19114	82.96±0.26
	CIP103575	82.97±0.43
	CIP107776	83.00±0.32
	ATCC19116	83.25±0.32
*L. welshimeri*	ATCC35897^T^	83.88±0.15
	019-3w	84.08±0.12

**Table 4:** Tm value of 33 strains which could not identify by *rarA* gene HRMA.

Strain no.	Tm (°C)	Identification results
31	83.06	*monocytogenes*
21	83.1	*monocytogenes*
76	83.12	*monocytogenes*
35	83.12	*monocytogenes*
23	83.12	*monocytogenes*
52	83.13	*monocytogenes*
12	83.13	*monocytogenes*
20	83.17	*monocytogenes*
11	83.18	*monocytogenes*
28	83.19	*monocytogenes*
22	83.2	*monocytogenes*
18	83.21	*monocytogenes*
25	83.21	*monocytogenes*
19	83.25	*monocytogenes*
46	83.64	*monocytogenes*
6	83.95	could not identify
13	84.04	*welshimeri*
16	84.12	*welshimeri*
2	84.14	*welshimeri*
61	84.2	*welshimeri*
4	84.2	*welshimeri*
7	84.2	*welshimeri*
14	84.22	*welshimeri*
10	84.24	*welshimeri*
9	84.25	*welshimeri*
1	84.26	*welshimeri*
38	84.27	*welshimeri*
5	84.3	*welshimeri*
37	84.32	*welshimeri*
17	84.35	*welshimeri*
15	84.42	*welshimeri*
3	84.8	*welshimeri*
45	85.56	*welshimeri*
