# Supplementary figures and images for: Establishment of a Simple and Rapid Identification Method for Listeria spp. by Using High-Resolution Melting Analysis, and Its Application in Food Industry
Source: PLoS One. 2014 Jun 11;9(6):e99223. doi: 10.1371/journal.pone.0099223 (PMC4053416; doi:10.1371/journal.pone.0099223)

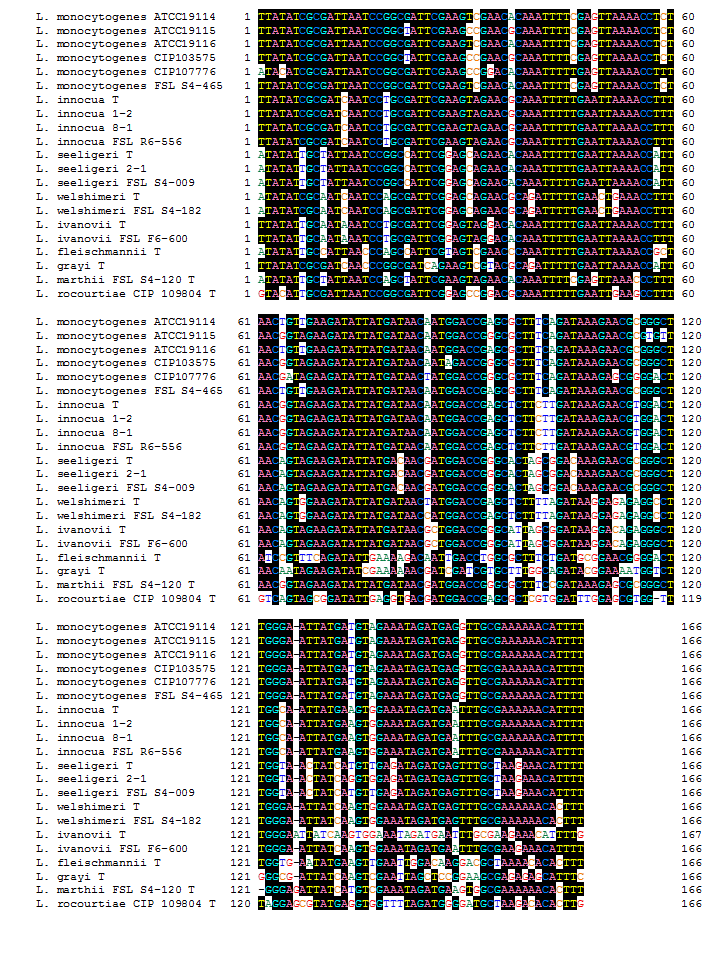

Supplement: Figure S1 — Alignment of partial sequences of rarA gene used for designing primer and analysis. The sequences of primer region are omitted. (TIF) [file pone.0099223.s001.tif]

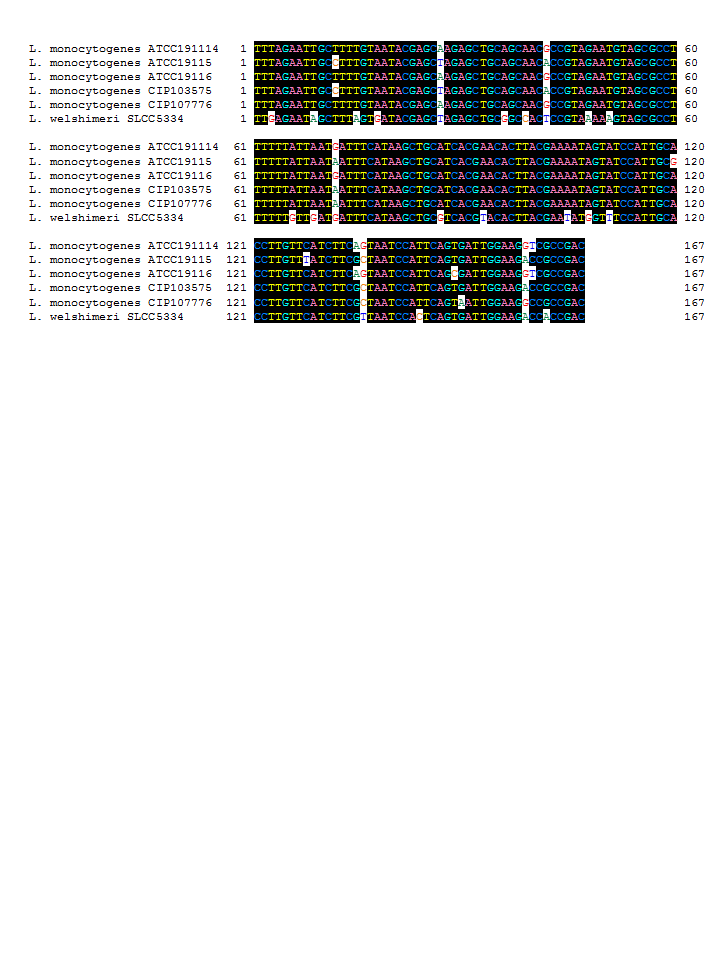

Supplement: Figure S2 — Alignment of partial sequences of ldh gene used for designing primer and analysis. (TIF) [file pone.0099223.s002.tif]
